# Structure of cellulose in birch phloem fibres in tension wood: an X-ray nanodiffraction study

**DOI:** 10.1186/s13007-023-01036-8

**Published:** 2023-06-17

**Authors:** Mira Viljanen, Sampo Muranen, Outi Kinnunen, Sebastian Kalbfleisch, Kirsi Svedström

**Affiliations:** 1 Department of Physics, University of Helsinki, (Gustaf Hällströmin Katu 2), P.O. Box 64, 00014 Helsinki, Finland; 2 Viikki Plant Science Centre, Institute of Biotechnology, University of Helsinki, (Viikinkaari 1), P.O. Box 65, 00014 Helsinki, Finland; 3 MAX IV Laboratory, Lund University, PO Box 118, S-221 00 Lund, Sweden; 4 Organismal and Evolutionary Biology Research Programme, Faculty of Biological and Environmental Sciences, University of Helsinki, 00014 Helsinki, Finland

**Keywords:** Scanning X-ray diffraction, Nanodiffraction, Tension wood, Phloem fibre, Cellulose microfibril, MFA

## Abstract

**Background:**

To gain a better understanding of bark layer structure and function, especially of the phloem fibres and their contribution to the posture control of trees, it is important to map the structural properties of these cells. The role of bark can also be linked to the reaction wood formation and properties which are essential when it comes to studying the questions related to tree growth. To offer new insights into the role of bark in the postural control of trees, we studied the micro- and nanoscale structures of the phloem and its nearest layers. This study is the first time, in which phloem fibres in trees have been extensively examined using X-ray diffraction (XRD).

We determined the orientation of cellulose microfibrils in phloem fibres of Silver birch saplings by using scanning synchrotron nanodiffraction. The samples consisted of phloem fibres extracted from tension, opposite and normal wood (TW, OW, NW).

**Results:**

Using scanning XRD, we were able to obtain new information about the mean microfibril angle (MFA) in cellulose microfibrils in phloem fibres connected to reaction wood. A slight but consistent difference was detected in the average MFA values of phloem fibres between the TW and OW sides of the stem. Using scanning XRD, different contrast agents (intensity of the main cellulose reflection or calcium oxalate reflection, mean MFA value) were used to produce 2D images with 200 nm spatial resolution.

**Conclusions:**

Based on our results, the tension wood formation in the stem might be related to the structure and properties of phloem fibres. Thus, our results suggest that the nanostructure of phloem fibres is involved in the postural control of trees containing tension and opposite wood.

**Supplementary Information:**

The online version contains supplementary material available at 10.1186/s13007-023-01036-8.

## Background

As higher plants, trees have developed xylem and phloem to take care of the mechanical stability of growth while maintaining the constant nutrient flow to all tissues. The strong support of the stem and branches is based on the fibre tissues with layered cell wall structures consisting of cellulose microfibril bundles ingrained into the hemicellulose-lignin matrix [29]. While xylem tissue is responsible for the mineral and water transport within the tree, phloem tissue is essential for the translocation of photosynthates and plant hormones. The inner stem structures consist of a periderm, phloem, vascular cambium and xylem. On the outermost stem structures of a tree, the secondary phloem is secured by the periderm which together form the outer bark layers. [39]. Cork cambium in the periderm, together with vascular cambium, is responsible for the secondary growth of the tree [20].

Located within the secondary phloem, the phloem sclerenchyma (phloem fibres) are known to be responsible for providing mechanical support for the other phloem tissues due to their secondary cell wall [11]. In trees and other plants [2], these fibres have been studied extensively using traditional light microscopy methods to provide insights into their structure on the cellular level. Besides this cellular level characterization, also the nanostructural properties of phloem have been characterized in the case of bast fibres (especially flax and hemp) and cotton [36, 26, 41]. To study the extremely thin phloem fibres in young tree saplings using X-ray diffraction, an X-ray beam with high intensity and at least micrometre level resolution is required to access the nanometre scale properties within these structures, underlining the necessity of the synchrotron experiments.

Moreover, not much is known about the properties of the cellulose microfibrils within the phloem fibres in trees, highlighting the importance of this study in providing insights into the structural properties of phloem fibres by X-ray scanning diffraction.

Bark has a central role in retaining the phloem nutrient transport and securing the innermost tissues from various threats, but it is also crucial for stem photosynthesis [1]. In some tree species, bark has also been found to have an important role in the postural control of trees [11]. Despite the importance and role of the various bark layers in the formation and securing of the wood tissues, the structures and dynamics of these structures have been characterized only scarcely.

Reaction wood is a special type of wood tissue generated in trees for their postural control due to various mechanical stimuli. Thus, reaction wood is found in all branches and the stem, when it is subjected to e.g., various wind loads, tilting or leaning. In deciduous trees, reaction wood is represented by tension wood (TW) on the upper side of the leaning stem, whereas tissue on the lower side is called opposite wood (OW) [28]. It has been observed that TW xylem tissues have different chemical compositions, and often distinct cellular and cellulose crystallite structures in comparison to OW and normal wood (NW) xylem [9, 18]. For example, in TW containing the G-layer (*gelatinous layer*), the cellulose content is often higher compared to NW [15]. The existence of TW without the G-layer has also been demonstrated in the literature [19].

The high cellulose content makes TW interesting for numerous applications, e.g., a source for a high lignocellulosic yield [3, 13], a solution for the recalcitrance problem [37] or potentially a source for nanocellulose production [22]. The function and structural properties of the bark layers on the TW side have only very recently been considered and characterized at the microscale [11].

The outstanding mechanical properties of wood are mainly explained by the properties and orientation of cellulose microfibrils [17, 24, 35]. These microfibrils are helically winded around the cell axis, forming an angle between the fibril and the axis called the microfibril angle (MFA). The determination of the MFA values based on the XRD data (the analysis of the azimuthal distribution of the crystalline cellulose reflections) was first established by Cave [8] and Meylan [30], and has since been developed further by many authors, e.g., by Terrill and Entwistle [14], Lichtenegger et al. [27] and Müller et al. [32]. It has been widely observed that in the TW xylem, the mean MFA values are smaller than the mean MFA in the NW xylem [10, 32]. Despite the extensive studies on MFA in reaction wood and NW xylem, there is no research conducted on the microfibrillar orientation in phloem fibres. Thus, it would be an important addition to the knowledge of cellulose microfibril properties to study the MFA in phloem fibres in TW to OW in comparison to NW phloem fibres.

To obtain information on bark layer structures, methods that provide high-resolution data on the nanoscale are required. Scanning X-ray nanodiffraction can be used to non-destructively map various materials in a two-dimensional grid with a highly focused X-ray beam. These measurements can provide details of the atomistic level order, composition and structures of the studied material, and the diffraction data can be used to image the structures of the sample on nano- and microscale [38].

One of the first scanning X-ray microdiffraction studies on wood was conducted by Lichtenegger et al. [27] in which they determined the MFA in adjacent cell walls in Norway spruce with a mesh scan using a synchrotron microbeam. Since then, microdiffraction has been used in several wood studies [33, 34],), and it has also been applied to further investigate the TW cellulose structure [10, 32]. With the recent emergence of the 4th generation brilliant and coherent synchrotron sources, the beam size is enabled to be down to the nanometer level, providing nanodiffraction experiments. However, there are only a few preliminary previous results considering nanodiffraction on wood samples, i.e. one previous study considered mainly the radiation damage of the FIB-prepared softwood sample [40].

In this work, we studied the nano- and microscale structures and orientation of cellulose microfibrils in bark and phloem layers of tilted and straight-control young silver birch saplings. The radial-longitudinally cut samples representing TW, OW and NW sides were studied using X-ray scanning nanodiffraction with 200 nm spatial resolution. The measurements were carried out using the NanoMAX beamline located at the MAX IV laboratory in Lund, Sweden. The research aimed to study the structural properties of the phloem fibres, acquire information on the cellulose microfibrils within these structures and the possible difference between TW, OW and NW. We hypothesise that there exists a possible difference between the mean MFA values of the phloem fibres on the TW side compared to the OW side, as detected in previous studies for wood xylem. The phloem fibres have an important role in the mechanical support of the stem and the MFA values are known to be strongly correlated with the mechanical properties of plant tissues. Thus, this hypothesis is linked to the role of tree bark in the postural control of the tree stems. There are no previous results on the MFA in the phloem fibres connected to wood, so, to the best of our knowledge, our results are the first evidence of the clear orientation of the cellulose microfibrils in the phloem.

## Results and discussion

**Fig. 1:**
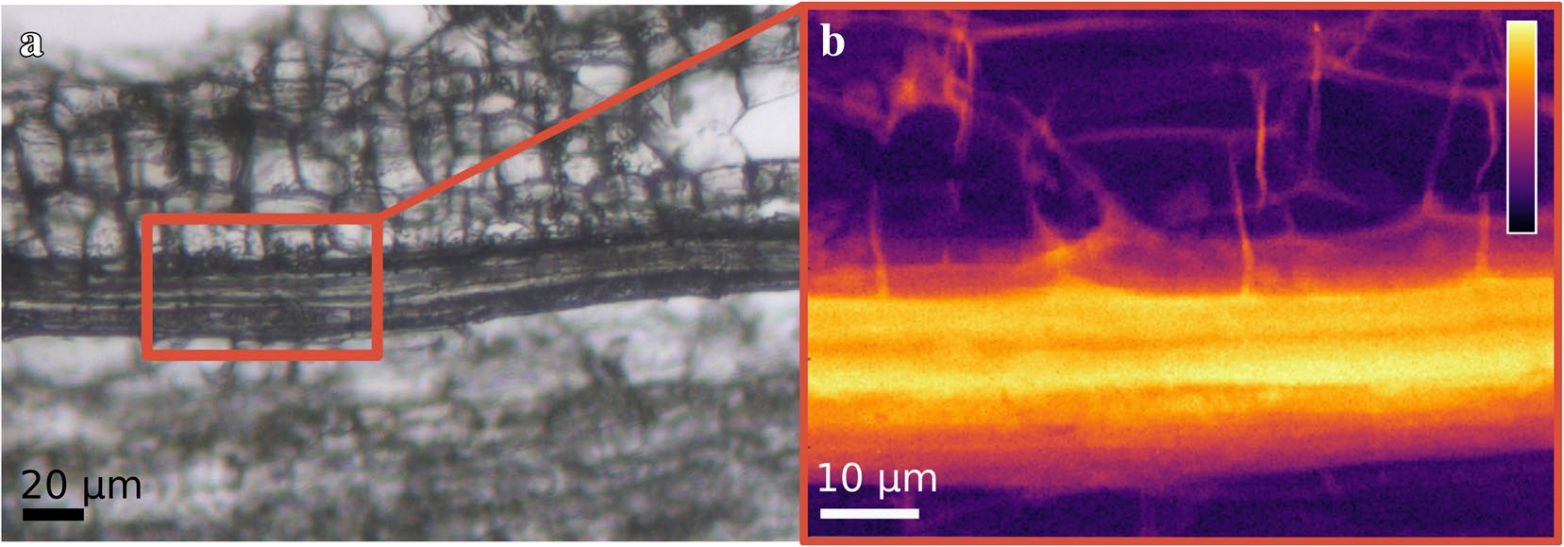
Visualisation of the measurement area in sample 9. A microscopy image of sample (**a**), showing the phloem fibres surrounded by other tissues. On right (**b**), the XRD image is constructed based on the intensity of the cellulose 200 reflection (i.e., the sum of the scattering intensities at q = 1.4–1.7 Å^−1^). The colour bar in image (**b**) represents the arbitrary intensity scale where the highest observed intensities are on white and the lowest on black. This same colour scale is used for all the XRD images representing the scattering intensities

An example of the studied samples is presented in Fig. 1, providing a visual example of the studied phloem fibres and the surrounding tissues by using light microscopy and scanning XRD.

### Microscopy

The TW presence in the sapling cross sections was confirmed by using the appropriate dying procedures (alcian blue 1% & safranin red 0.05%) and light microscopy imaging.

**Fig. 2:**
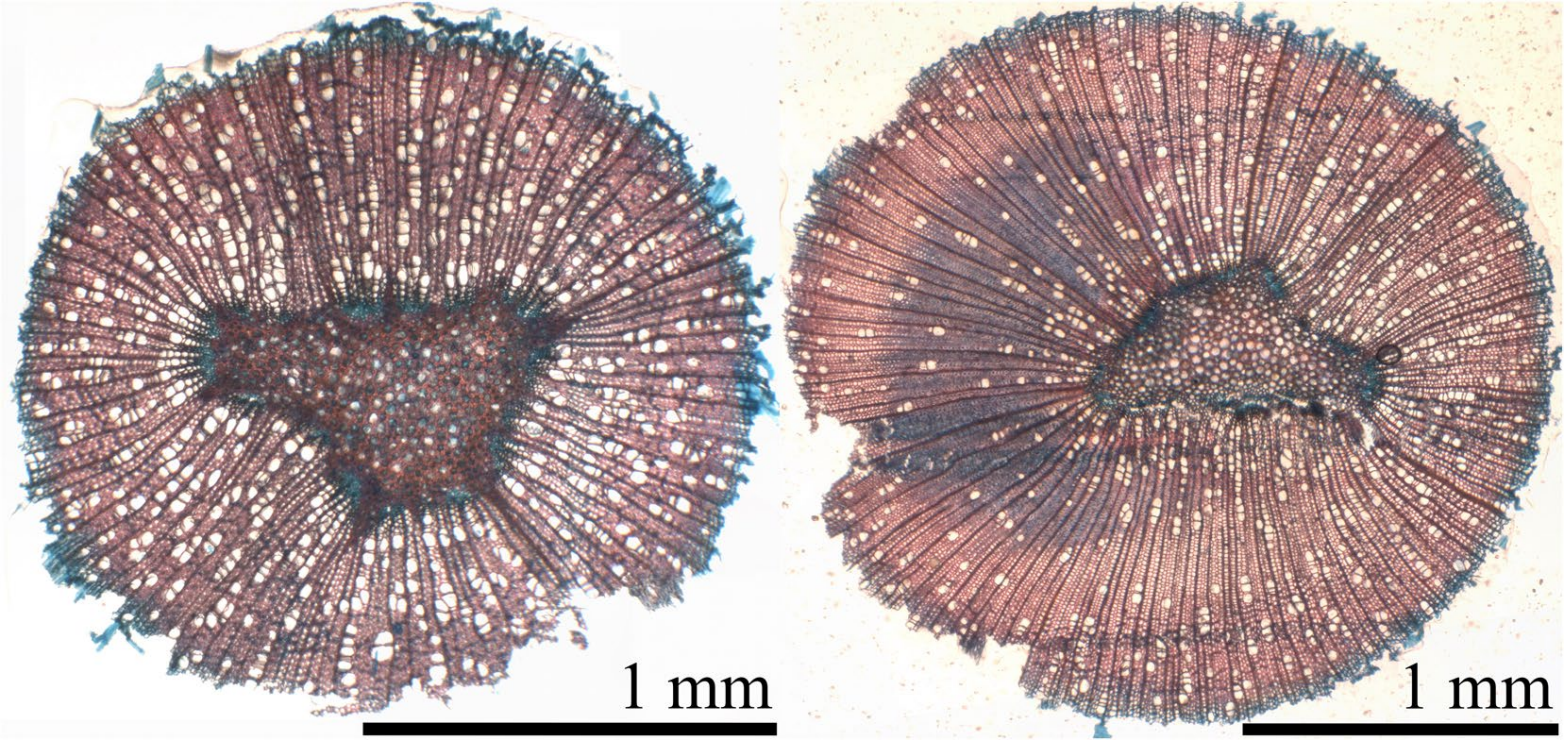
Stained cross sections of reference sample 1 on the left and tilted sample (sample 16) on the right. On the tilted sample, the TW formation can be seen to cover almost all of the left side of the xylem of the cross-section. A similar TW formation is absent in the reference sample

In Fig. 2, examples of the dyed slices of reference and tilted samples are presented. The presence of TW is observed in sample 16 (see Fig. 2, right image) as indicated by the large blue stained (xylem) area. Similar stained areas were observed also in 5/6 of the other tilted samples.

The frequency of the vessel elements was also lower on the TW side compared to the OW side within the tilted samples, which has been detected to be a feature of TW (e.g. [4]). Thus, the overall amount of vessel elements in the tilted samples was also lower compared to the NW samples.

### Scanning XRD

As can be observed from Fig. 1, XRD can be used to visualise and identify similar structures that are visible by microscopy, and in addition, all the parameters that can be determined by XRD data give information on the nanoscale structural properties.

**Fig. 3:**
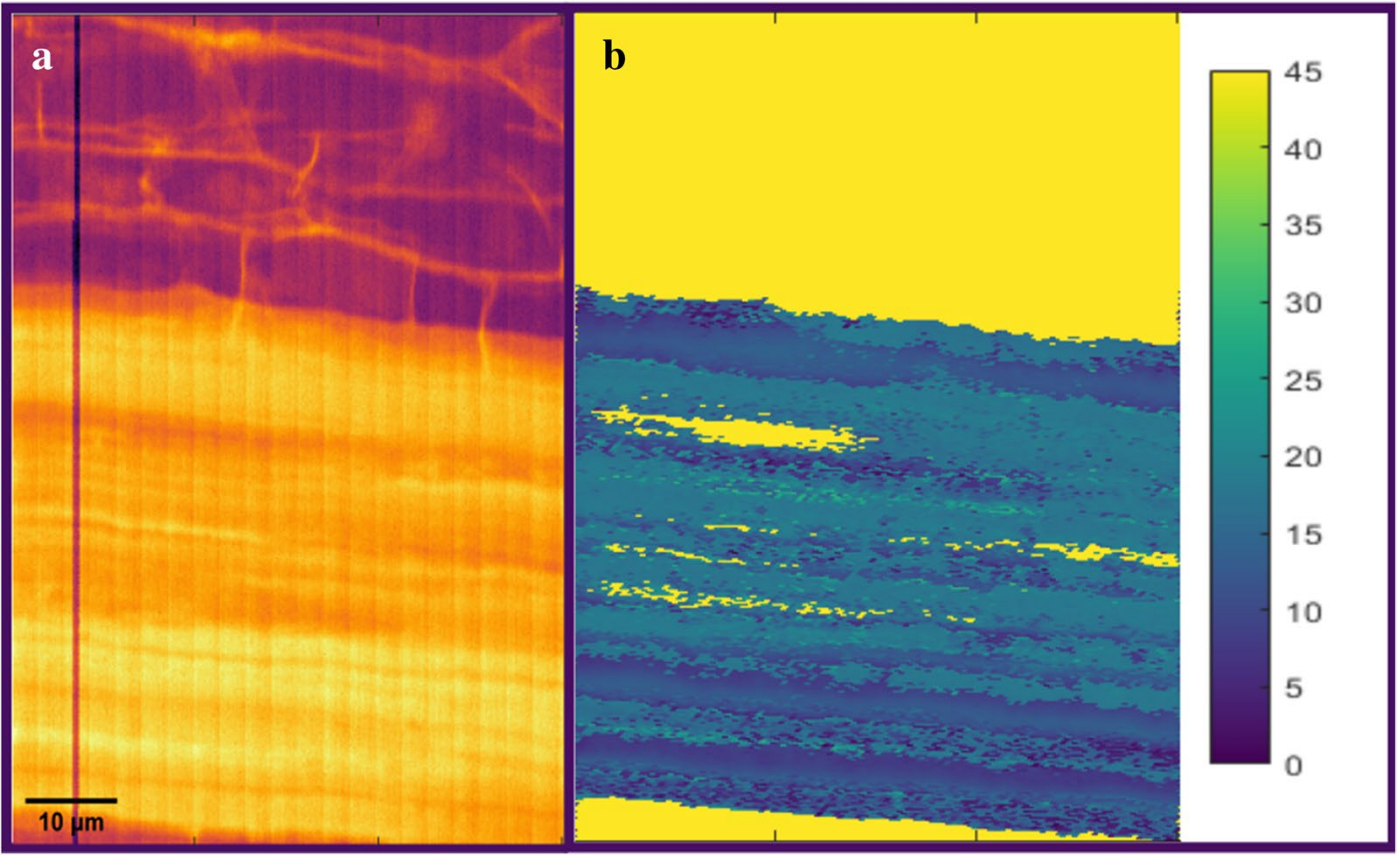
Visualisation of phloem fibres by using (**a**) the cellulose 200 reflection intensity vs. (**b**) the mean microfibril angle values in sample 16. In (**a**), the XRD data given is constructed of the highest intensity around the cellulose 200 reflection (q = 1.4–1.7 Å^−1^). In (**b**), the visual presentation of the same sample area is constructed by using the microfibril angle value. The units of the MFA values are in degrees. The bright yellow colour (45 degrees) in (**b**) does not correspond to a real MFA value (i.e., it represents the background). The length scalebar is the same for both the pictures

Figure 3 presents the XRD images with two different contrast: (A) the intensity of the cellulose 200 reflection, and (B) the mean MFA values. The highest observed scattering intensities of the cellulose main reflection (200) corresponded to the areas covered by phloem fibres (Figs. 1 and 3).

The overall thickness of the phloem fibres area in all samples varied between 15 µm and 75 µm without any systematic differences between TW and OW samples. In Fig. 1b and Additional file 1: Figure S2B, within the cellulose-rich areas (the zones with the highest relative scattering intensities on bright yellow/white colour), striped, micrometre-level structures originating from crystalline cellulose can be observed. Due to the intensity value corresponding directly to the amount of crystalline cellulose in that pixel area, it can be said that these stripe features arise from the differences in the cellulose contents. The differences in the crystalline cellulose contents might be caused by several factors (e.g., variation in thickness and/or crystallinity of the fibres). These different factors might be connected to further hierarchy within the phloem fibres, potentially indicating differences in the secondary cell wall formation between different cells.

**Fig. 4:**
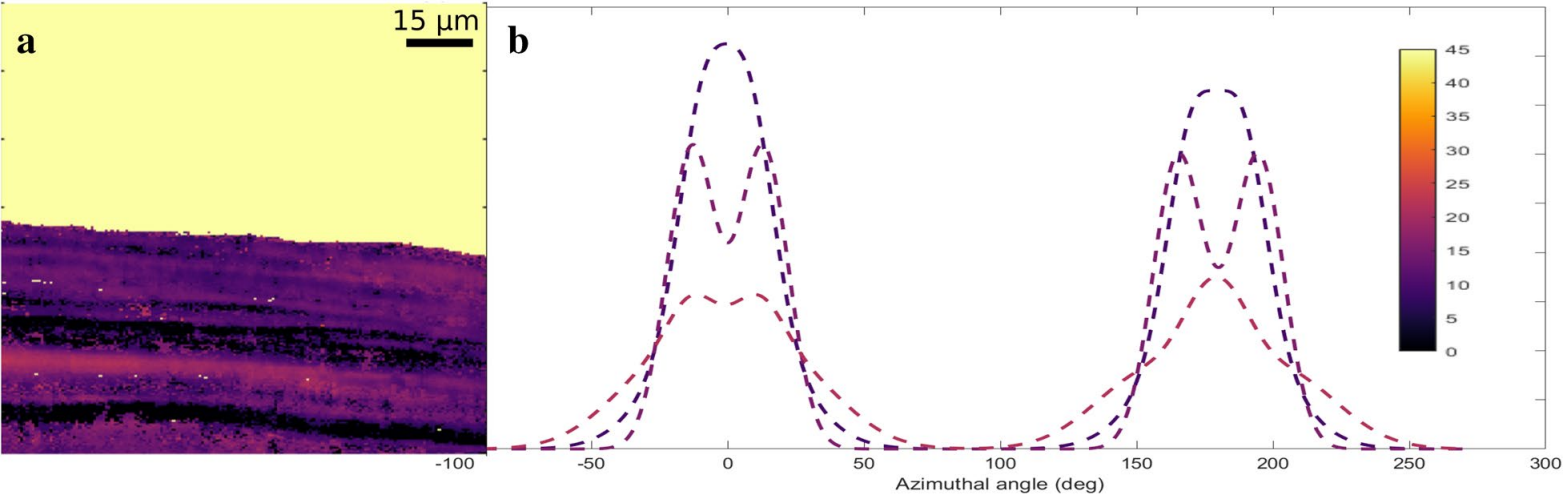
Visualisation of (**a**) the mean MFA values vs. (**b**) typical fitted azimuthal profiles of selected areas in sample 1. The colouring of the azimuthal profile curves corresponds to the colours in the MFA value data on the left. The light-yellow colour (45 degrees) in (**a**) does not correspond to any real MFA value

For the cellulose-rich areas within the phloem fibre regions, the MFA analysis was conducted (see Fig. 4). The average values of all the mean MFA values inside these areas are summarized in Table 1 for all the samples. In the TW samples, the obtained average of the mean MFA values was 14 ± 2 degrees while for the OW samples, the value was 18 ± 2 degrees. This slight difference in the MFA values between TW and OW was systematic throughout all the samples and was detectable by the differences in the azimuthal profiles (see Additional file 1: Figure S3). However, for the reference NW samples, the obtained MFA values were similar or slightly lower than in the TW samples, the average of the mean MFAs of the NW samples being around 11 ± 2 degrees.

**Table 1:** Microfibril angle values per each sample with the indication of which side the sample was from. Reference samples are marked as REF, opposite wood as OW and tension wood as TW. Sample pairs 9 and 10 and 11 and 12 are the TW + OW sides that have been extracted from the same sapling

Sample#	MFA ± 2 (°)	Side
1	10	REF
2	12	REF
4	11	REF
6	14	OW
8	20	OW
9	13	TW
10	19	OW
11	17	TW
12	18	OW
14	18	OW
16	13	TW

The difference in MFA between normal wood and reaction wood in the xylem in both hard- and softwood species is widely reported in the literature. In TW, the mean MFA of xylem fibres is known to be smaller than the mean MFA in NW xylem fibres, and this difference has also been observed previously using various XRD techniques [10, 21, 32]. In our study, the opposite observation was made: we obtained smaller MFA values for the reference NW samples in comparison to the TW side samples. This observation could be explained by the small number of sample stems and by the extremely young age of the samples in the study. It must also be noted that the MFA values were determined for the cells representing the most juvenile cells, i.e., the phloem cells. The direct comparison of the MFA results between various trees grown in various conditions is not unambiguous due to the possibility of significant structural differences between the individual juvenile stems. Thus, the comparison between the TW and OW side properties of the same individual tree is the most justified approach, making our observations on the clear differences between the two sides within the same tilted tree reliable.

There are only a few studies regarding structural properties in bark layers related to TW formation [11], and this is the first time MFA has been specifically examined in phloem fibres in the case of TW.

The mean MFA of plant fibres has a close connection to the mechanical properties: this has been observed in wood xylem [24], but also in phloem fibres of flax [5], hemp and sisal [6] and cotton [31]. The very low MFA’s (close to 0°) in TW samples have been connected to the property of the G-layers [10]. Thus, the various MFA values indicate different mechanical properties. The bark structures, including phloem fibres, and their role in the posture control of a tree has been discussed just recently: Clair et al. studied the curvatures of the tilted saplings after their release from the tilted position and after removing the bark, and observed that the bark had contribution in straightening of the stems, stating that the bark contribution arises from the organized trellis structure in the inner bark [11]. Our results on the difference in the MFA values between TW and OW side phloem fibres might be connected to these observations, and thus support the discussion on the role of tree bark in the postural control of the stems.

The total scattering intensity was significantly lower in all the other parts of the image (i.e. in the tissue around the phloem fibres) compared to the scattering from the cellulose in phloem fibres (see Additional file 1: Figure S4). This is most likely due to the small amount and nature of the samples: the sample thickness was only 50 µm at maximum, and the samples were prepared from extremely young saplings with thin cell walls.

It was also observed that the intensities of the collected radial scattering data were low (see Additional file 1: Figure S5), making it impossible to determine the FWHM of the cellulose 200 reflection and conduct thus enough reliable analysis of the cellulose crystallite widths.

One possible explanation for this observation could be the low crystallinity of the cellulose so that the secondary cell wall in the tissues of phloem fibres has not been fully developed yet. Clair et al. studied the cellulose reflections (200 & 004) along a development sequence in poplar using an X-ray microbeam line scan [10]. They observed a clear scattering signal from 200 reflection in the phloem fibres zone, but they did not analyse that signal any further in their study. Instead, they focused on the scattering results related to the zones after the cambial area. They observed that the scattering signal was low within and near the cambial zone structures and explained that to be due to the absence of the secondary cell wall. This means that near the cambium, the amount of crystalline cellulose within these structures is generally very low compared to xylem tissue, thus leading to a very low scattering signal from the cellulose microfibrils.

Another possible explanation leading to low scattering intensity in biological wood samples is the potential radiation damage. Storm et al. [40] studied the effects of radiation damage on FIB-treated softwood samples using scanning X-ray nanodiffraction and they showed that the observed scattering significantly weakens due to secondary radiation damage between subsequent measurement spots in a line scan. We did not observe similar weakening of the collected X-ray signals in any of the recorded scattering patterns between consecutive measurement points during the radiation tests or actual measurements. Storm et al. also concluded that the FIB treatment played a role in the observed radiation damage, which was not used in our study. Thus, it is unlikely that the radiation damage is the reason for the low scattering signal of the cellulose reflections.

**Fig. 5:**
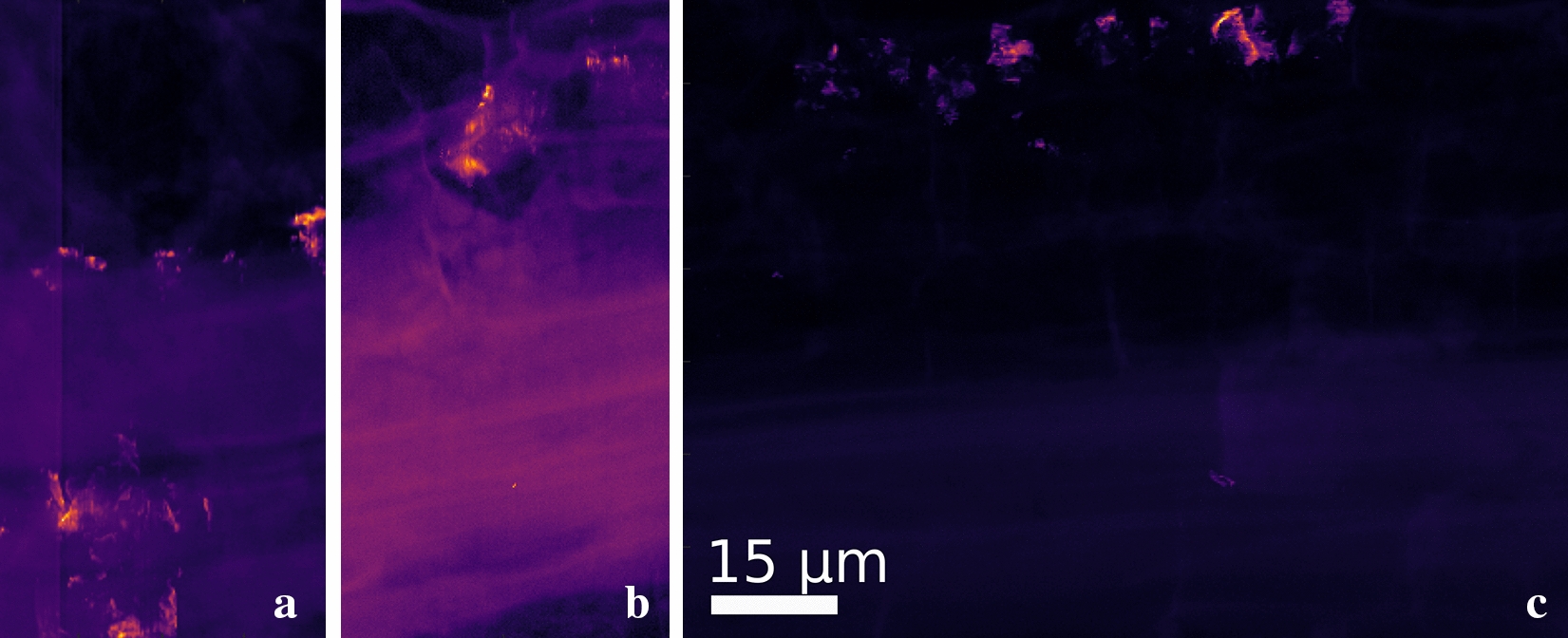
Examples of detected areas corresponding to calcium oxalate crystals in samples 4 (**a**), 6 (**b**) and 12 (**c**). The scattering data was selected on the specific q-scale area (1.0–1.15 Å^−1^), covering the calcium oxalate reflections. The scalebar is the same for all the pictures

**Fig. 6:**
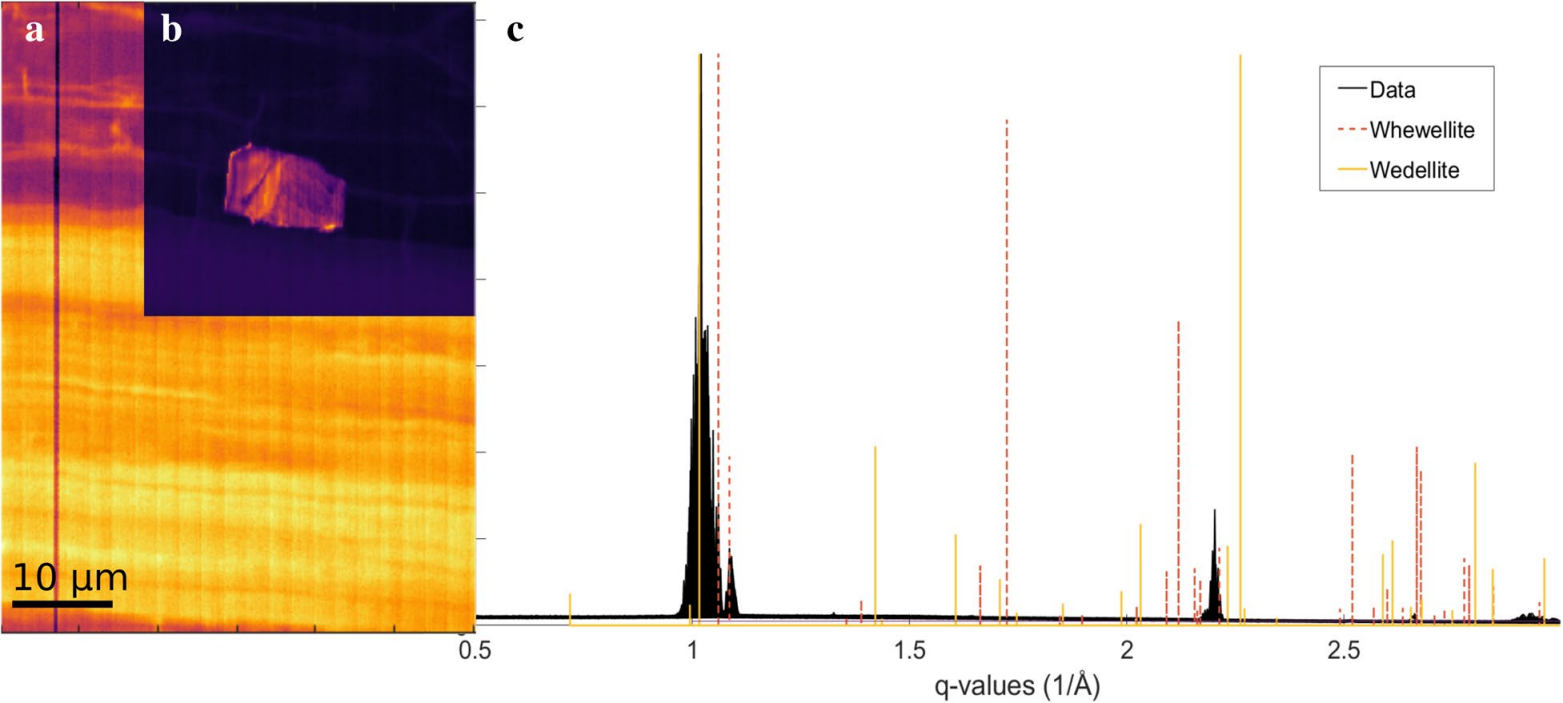
Example of the detected calcium oxalate in sample 16. On the left (**a** and **b**), the known reflections of whewellite (CaC_2_O_2_) and weddellite (CaC_2_O_4_) were used to construct the scattering data on the selected q-scale (1.0–1.15 Å^−1^). On the right (**c**), is the scattering data of sample 16 with the reflections of whewellite and weddellite which shows the crystal contains these compounds

In some of the samples, based on the XRD images, scattering from non-cellulosic materials was observed within q-ranges q = 1.05–1.15 Å^−1^ and q = 2.1 -2.2 Å^−1^. This can be seen in Figs. 5 and 6, which both contain areas with high scattering intensities from reflections from non-cellulose origin. These diffraction maxima correspond well to the reflections of calcium oxalate compounds, whewellite (CaC_2_O_2_ · H_2_O) and weddellite (CaC_2_O_4_ · H_2_O), which are known to be present in plant tissues. The crystal-containing idioblast cells in various plant tissues are thought to function as herbivore deterrents and as storage deposits for excess calcium, and in some cases even provide extra support as tissue stiffeners [12].

The shapes, dimensions and amounts of the detected areas of the CaO crystallites were varying; for most of these areas, the dimensions were on average in the range of 5–8 µm. In sample 16 (Fig. 6b), a larger area was detected (approximately 8.5 × 15 µm). These dimensions correspond well to an excretory idioblast cell, containing the above-described substances [16]. The observed intensities of the calcium oxalate compounds were neither uniform nor arising from the same reflections (Fig. 5), suggesting that the crystals consist of mosaic-like, polycrystalline structures. These structures could potentially be similar to the box-like, raphide bundles observed beneath the stem epidermis in *Dieffenbachia seguine* by [12]. This suggestion is further supported by the note made by the same authors as the box bundles are often present near those structures that benefit from the extra support [12].

## Conclusions

We studied the phloem fibres and near-bark structures using synchrotron X-ray nanodiffraction in young, tilted silver birch saplings. For the first time, the structure and orientation of cellulose microfibrils in phloem fibres in tension and opposite wood sides were determined by using this novel technique. These first observations would suggest, that the microfibril angle in the phloem fibres on the tension wood side was systematically smaller compared to the phloem fibres on the opposite wood side. The MFA value in phloem fibres on the tension wood side was approximately 14 ± 2 degrees while in the corresponding location in the opposite wood, it was approximately 18 ± 2 degrees. These results would support the hypothesis that the bark and phloem layer structures are also involved in the postural control of the trees, but this very first observation on phloem structural properties needs additional studies for furthering the biological conclusions.

Furthermore, we were able to observe scattering from non-cellulosic structures, i.e., from calcium oxalate deposits associated with the phloem fibres and bark structures. The shapes and sizes of these deposits varied throughout the samples, corresponding to the idioblast cell dimensions.

To conclude, scanning nanodiffraction is well suited for the characterization method for mapping the structural properties of the phloem and other bark layers. Thus, we foresee the further impact of this research method to be highly significant for the field in the very near future.

## Methods

### Materials

To study the bark layer structures, Betula pendula saplings were cultivated. Silver birch (Betula pendula) plants (bought as a dormant sapling from Taimi-Tapio) were planted in 1.5 l pots with a soil mixture of peat, sand and vermiculite (in 6:2:1 respectively). Dormancy was broken for 1.5 weeks (22.-3.3.2020). Six of the plants were placed in a tilted stand (~ 45 degrees) and 4 control plants were grown straight from 3.3.2020 onwards. Plant heights were as indicated below on 3.3.2020. Plants were grown under ambient light conditions (HPS 18/6 added light), + 20 °C, in a greenhouse. The plants were sampled after 40 days of growth on 12.4.2020, photographed and stored at −20 °C.

**Fig. 7:**
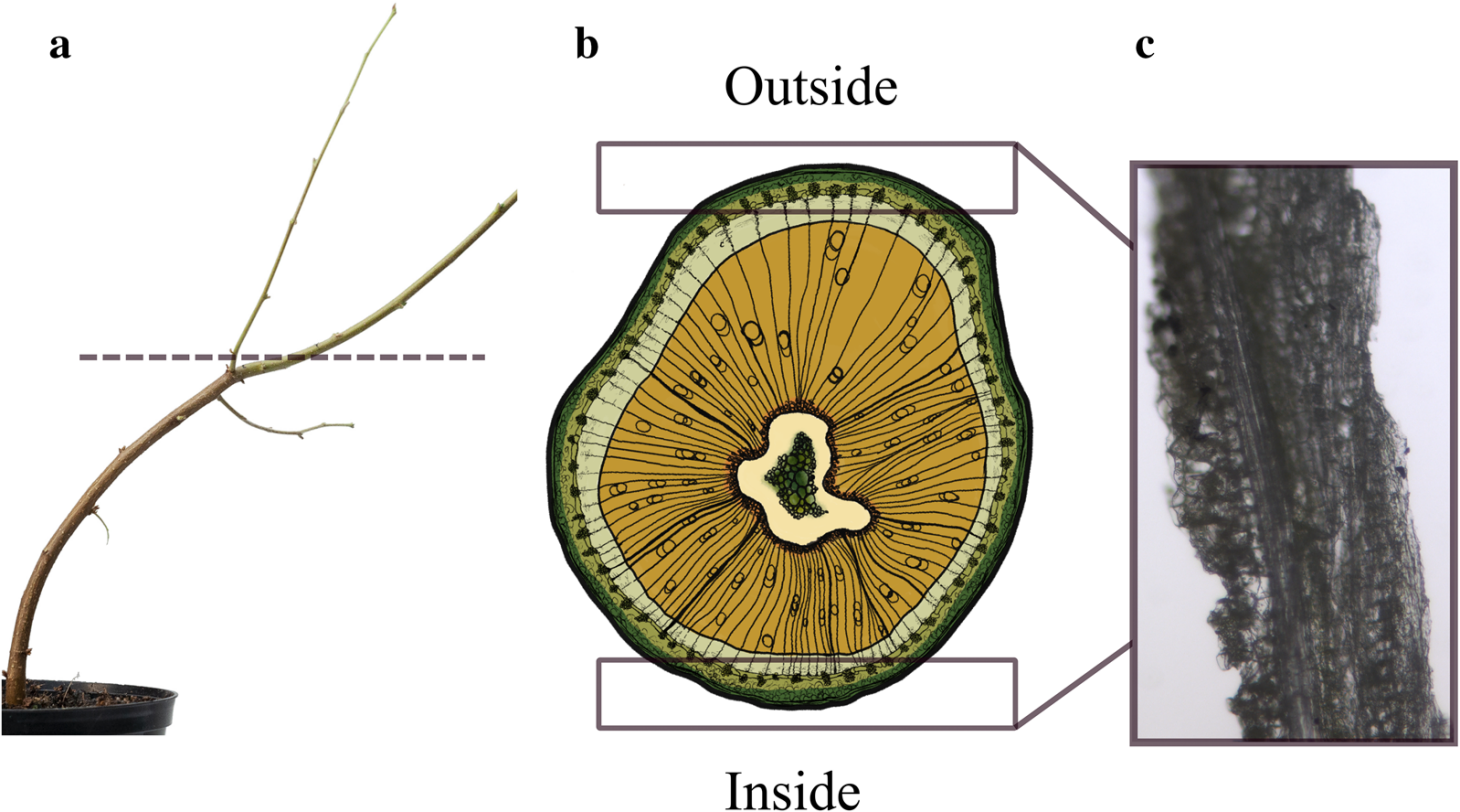
Example of the sample extraction locations. In (**a**) the dotted line marks the height where the cross-sections (**b**) were extracted, and the boxes in (**b**) mark the areas where the samples (**c**) were cut from

Similar regions as indicated in the image (Fig. 7a and b) were used for cross sections and extraction of bark. The bark samples were then cut into 1 cm round pieces from carefully selected spots, halved in the vertical directions. These pieces were then further sectioned radial-longitudinally into around 30 µm thick slices using a cryotome (Leica CM3050 S). The slices were divided into two, and the other half was stained with safranin (0.05% safranin in 50% EtOH) and examined with a microscope (Leica DMLB equipped with DFC490) using the fluorescence contrast mode to verify the presence of lignified phloem fibres and bark tissues. If the presence was confirmed, the unstained half was then sealed between two Si-membranes (Norcada, window size 1 mm x 1 mm). As a final step, the membranes were glued into the sample holders provided by the NanoMAX beamline.

A separate set of cross-sections of the studied samples was prepared to study the presence of TW within the samples. The cross-sections were obtained approximately 2 cm away from the samples made for NanoMAX measurements. The cross-sections (50 µm) were prepared using cryotome and after stained with alcian blue (1% alcian in H_2_O) and safranin red (0.05% safranin in 50% EtOH). Images from the stained slices were captured with a microscope (Leica DMLB equipped with DFC490) and constructed into a full field image of each slice in Photoshop (v.23.2.2) using the automated photo merge tool with default settings.

### Experimental: scanning X-ray nanodiffraction

The cellular structures and microfibril angles were studied using scanning X-ray nanodiffraction. The diffraction measurements were conducted at the MAX IV facility at the NanoMAX beamline. A detailed description of the used measurement setup is presented in [7, 23].

For the measurements, perpendicular measurement geometry was used with a beam energy of 12 keV corresponding to the wavelength of 1.03 Å. To collect the scattering data, Pilatus2 1 M detector was used. The detector was placed at a distance of 177.5 mm from the radiation source and calibrated for the corresponding q-values (the length of the scattering vector) using the Si-sample. The q-range of the measurements was 0.1–3.0 Å^−1^.

Cryo-cooling of the samples during these measurements was not possible, thus careful radiation damage tests were conducted to estimate the optimal exposure time and minimum beam damage on the samples.

Before the diffraction measurements, the attached optical sample microscopes were used to determine the measurement area of interest for each sample. The chosen areas were approximately 40–80 µm depending on the sample. The diffraction data of the chosen areas were collected using a beam size of 100 nm with 200 nm step size in both x- and y-directions of the beam, resulting together 120-150 k scattering patterns collected per sample. Based on the radiation tests, the exposure time for each measurement point was chosen to be 0.1 s, resulting in an average of 2.5 h of measurement time for each sample.

### Data-analysis

The acquired data were integrated using the open-source, Python-based software *PyFAI* [25]. Before integration, averaging, normalization, and masking procedures were applied to the data.

For each scattering pattern, the integration was done in both radial and azimuthal directions. The radial integration was conducted on the whole 2D pattern with 1000 points in the q-range of 0.5–3.0 Å^−1^. The azimuthal integration was limited around the cellulose 200-reflection to acquire the azimuthal profiles for the MFA analysis. Thus, the azimuthal integration was conducted from -180 to 179 degrees with 1-degree steps by integrating the q-scale 1.4–1.6 Å^−1^. To improve the statistics, the data were summed over three subsequent scan points (in the parallel direction) of the fibre.

The microfibrillar orientation analysis and visualisation of the XRD data were conducted using MATLAB. The MFA was analysed using the method explained in detail in [34]. This method is directly based on the application of the equation formulated by Cave [8], which in this case was used to connect the azimuthal profile of the cellulose 200 reflection and the MFA. The method presumes each cell to have a rectangular cross-sectional shape with 4 cell walls contributing to the azimuthal intensity per peak. The cell wall contributions were fitted to the full azimuthal profile using 8 Gaussians with peak widths and intensities chosen as fitting parameters (see examples in Additional file 1: Figure S3c and d). Other fitting parameters included the position of the centre of the azimuth, the rotation of the cell walls with respect to the direct beam and the intensity sum between the Gaussians fit on the left and right peak due to detector geometry.

The XRD images were generated by a custom script with varying contrast agents: total scattered intensity, the intensity of cellulose 200 reflection and the intensities of the chosen calcium oxalate reflections. From these images, different structural features (e.g. average sizes of the calcium oxalate crystals and phloem fibre thicknesses) were observed.

## Supplementary Information

**Additional file 1: Table S1.** List of samples cut from a specific height of the plant. Samples from #6 to #16 are from plants grown in a tilted position. Height resembles the original height of the plant when the experiment started (growing in a tilted position) and the minimum height from which the newly formed phloem fibre samples were collected. **Figure S1.** Examples of the cultivated plants, a) an example of a straight plant as a source for the reference samples., (b) an example of a tilted plant as a source for the TW/OW samples. The tilted plant was grown at a 45-degree angle. The black bar in both images is 20 cm. **Figure S2.** Visualisation of the measurement area in sample 1. Microscopy image of the sample on left (a). On right (b), the XRD data constructed by the maximum intensity around the cellulose 200 reflection (q = 1.4–1.7 Å^−1^). The colour scale bar on the right image represents the intensity scale where white corresponds to the highest relative intensity and black to the lowest. **Figure S3.** Examples of the normalized azimuthal profiles (printed every 500^th^ of the data points) in TW vs OW. In a), the azimuthal profiles of TW in sample 9 are presented. For comparison, in b), the azimuthal profiles of OW in sample 10 are presented. Images c) and d) represent the MFA fits on the azimuthal data for the data points 184 in sample 16 (mean MFA = 7.9°) and 14397 in sample 6 (mean MFA = 14.5°), respectively. The contributions from the 4 different cell walls (CW) in a rectangular cell have been used as the basis of the fits (BF = back cell wall (CW), FF = front CW, RF = right CW, LF = left CW). **Figure S4.** Visualisation of the XRD data of the total intensities (q = 0.5 – 3.0 Å^−1^) in sample 2.). The colour scale bar represents the intensity scale where white corresponds to the highest relative intensity and black to the lowest. **Figure S5.** Examples of the radial profiles (the full 2D pattern integrated without any corrections in the data and printed every 1000^th^ of the data points) in TW vs OW. In (a), the radial profiles of TW in sample 11 are presented. For comparison, in (b), the radial profiles of OW in sample 12 are presented. Spike around q = 2.5 Å^−1^ arises from the used equipment and does not represent sample data.

## Data Availability

The datasets used and analysed during the current study are available from the corresponding author upon reasonable request.

## Abbreviations

XRD: X-ray diffraction
TW: Tension wood
OW: Opposite wood
NW: Normal wood
MFA: Microfibril angle
FWHM: Full width at half maximum
FIB: Focused ion beam
