# Supplementary material for: Structure of cellulose in birch phloem fibres in tension wood: an X-ray nanodiffraction study
Source: Plant Methods. 2023 Jun 17;19:58. doi: 10.1186/s13007-023-01036-8 (PMC10276511; doi:10.1186/s13007-023-01036-8)
Supplement: Supplementary file 1 — Additional file 1: Table S1. List of samples cut from a specific height of the plant. Samples from #6 to #16 are from plants grown in a tilted position. Height resembles the original height of the plant when the experiment started (growing in a tilted position) and the minimum height from which the newly formed phloem fibre samples were collected. Figure S1. Examples of the cultivated plants, a) an example of a straight plant as a source for the reference samples., (b) an example of a tilted plant as a source for the TW/OW samples. The tilted plant was grown at a 45-degree angle. The black bar in both images is 20 cm. Figure S2. Visualisation of the measurement area in sample 1. Microscopy image of the sample on left (a). On right (b), the XRD data constructed by the maximum intensity around the cellulose 200 reflection (q = 1.4–1.7 Å−1). The colour scale bar on the right image represents the intensity scale where white corresponds to the highest relative intensity and black to the lowest. Figure S3. Examples of the normalized azimuthal profiles (printed every 500th of the data points) in TW vs OW. In a), the azimuthal profiles of TW in sample 9 are presented. For comparison, in b), the azimuthal profiles of OW in sample 10 are presented. Images c) and d) represent the MFA fits on the azimuthal data for the data points 184 in sample 16 (mean MFA = 7.9°) and 14397 in sample 6 (mean MFA = 14.5°), respectively. The contributions from the 4 different cell walls (CW) in a rectangular cell have been used as the basis of the fits (BF = back cell wall (CW), FF = front CW, RF = right CW, LF = left CW). Figure S4. Visualisation of the XRD data of the total intensities (q = 0.5 – 3.0 Å−1) in sample 2.). The colour scale bar represents the intensity scale where white corresponds to the highest relative intensity and black to the lowest. Figure S5. Examples of the radial profiles (the full 2D pattern integrated without any corrections in the data and printed every 1000th of the [file 13007_2023_1036_MOESM1_ESM.docx]

Table S1. List of samples cut from a specific height of the plant. Samples from #6 to #16 are from plants grown in a tilted position. Height resembles the original height of the plant when the experiment started (growing in a tilted position) and the minimum height from which the newly formed phloem fibre samples were collected.

| Sample | Height (cm) |
| --- | --- |
| #1, #2 & #4 | 26 |
| #6 | 16,5 |
| #8 | 19,5 |
| #9 & #10 | 18 |
| #11 & #12 | 15,5 |
| #14 | 23 |
| #16 | 20,5 |


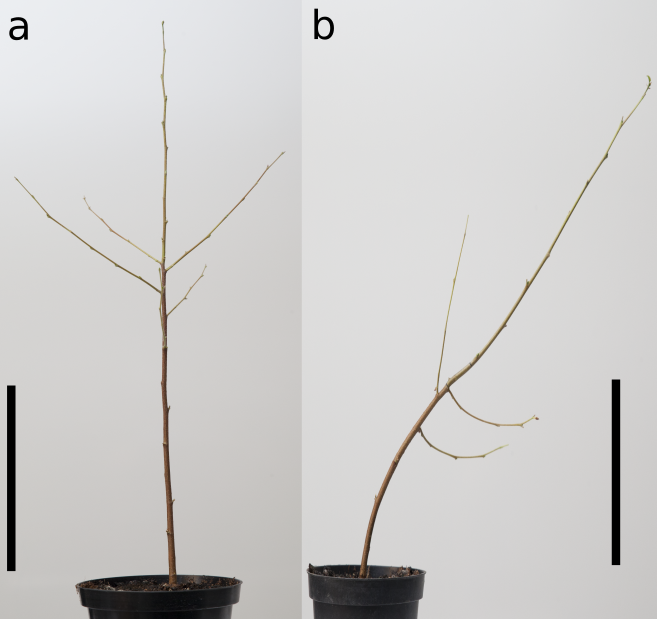


Figure S1. Examples of the cultivated plants, a) an example of a straight plant as a source for the reference samples., (b) an example of a tilted plant as a source for the TW/OW samples. The tilted plant was grown at a 45-degree angle. The black bar in both images is 20 cm.

*
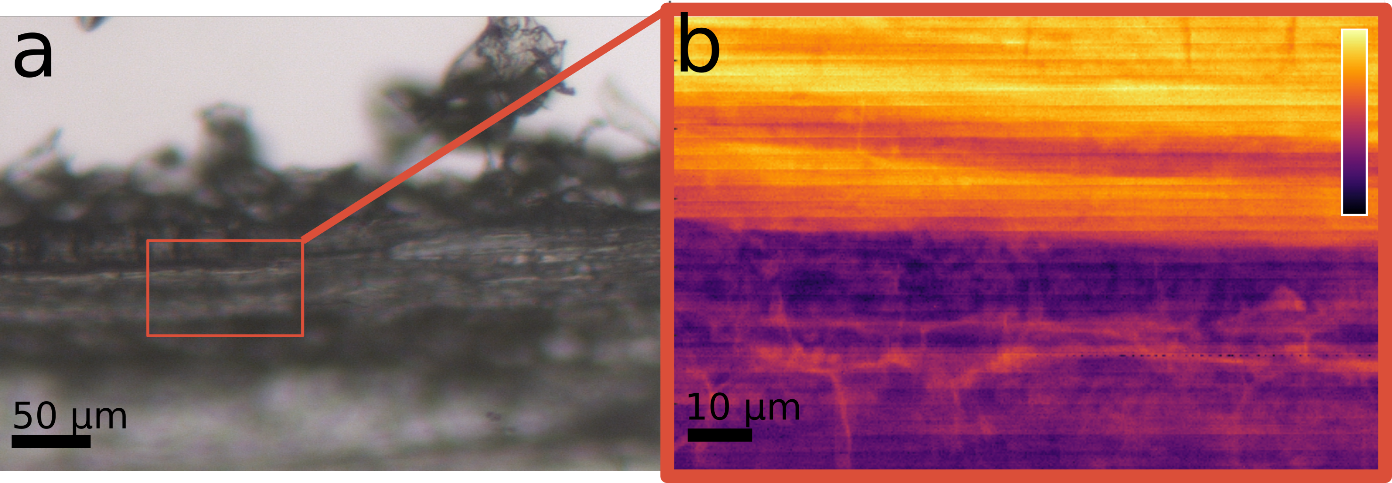
*

Figure S2. Visualisation of the measurement area in sample 1. Microscopy image of the sample on left (a). On right (b), the XRD data constructed by the maximum intensity around the cellulose 200 reflection (q= 1.4-1.7 Å^-1^). The colour scale bar on the right image represents the intensity scale where white corresponds to the highest relative intensity and black to the lowest.


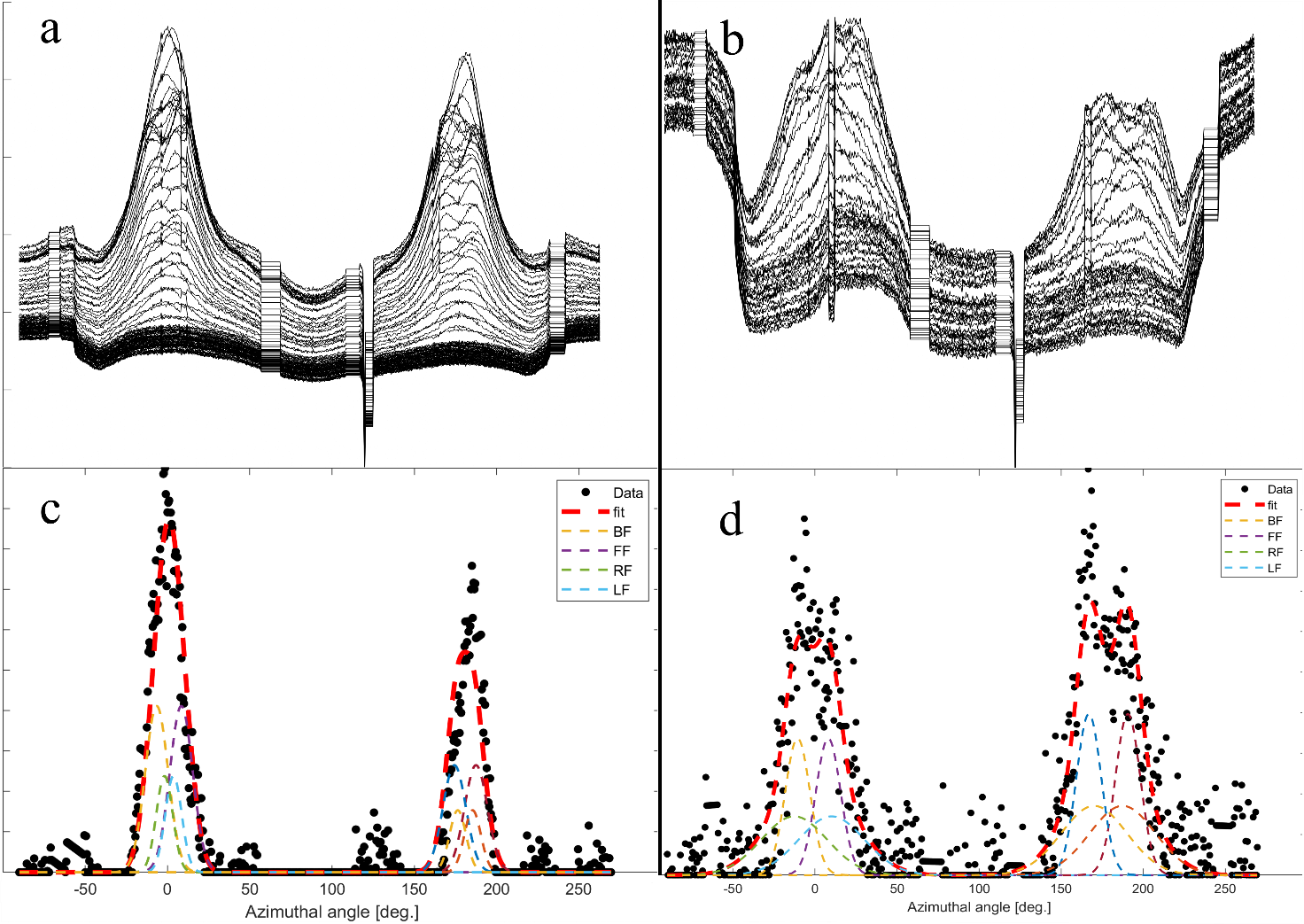


Figure S3. Examples of the normalized azimuthal profiles (printed every 500^th^ of the data points) in TW vs OW. In a), the azimuthal profiles of TW in sample 9 are presented. For comparison, in b), the azimuthal profiles of OW in sample 10 are presented. Images c) and d) represent the MFA fits on the azimuthal data for the data points 184 in sample 16 (mean MFA = 7.9⁰) and 14397 in sample 6 (mean MFA = 14.5⁰), respectively. The contributions from the 4 different cell walls (CW) in a rectangular cell have been used as the basis of the fits (BF=back cell wall (CW), FF=front CW, RF=right CW, LF=left CW.)


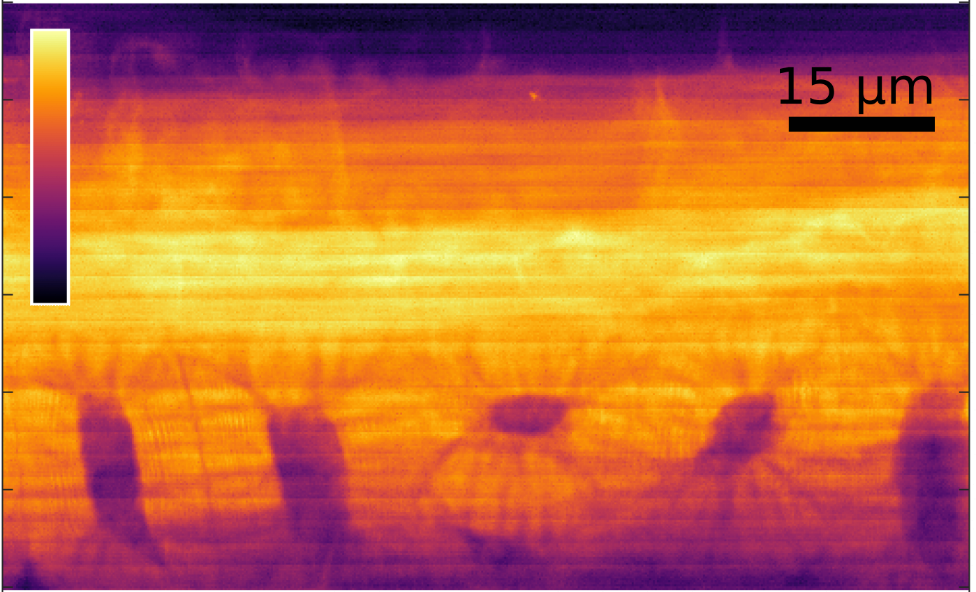


Figure S4. Visualisation of the XRD data of the total intensities (q= 0.5 – 3.0 Å^-1^) in sample 2.). The colour scale bar represents the intensity scale where white corresponds to the highest relative intensity and black to the lowest.


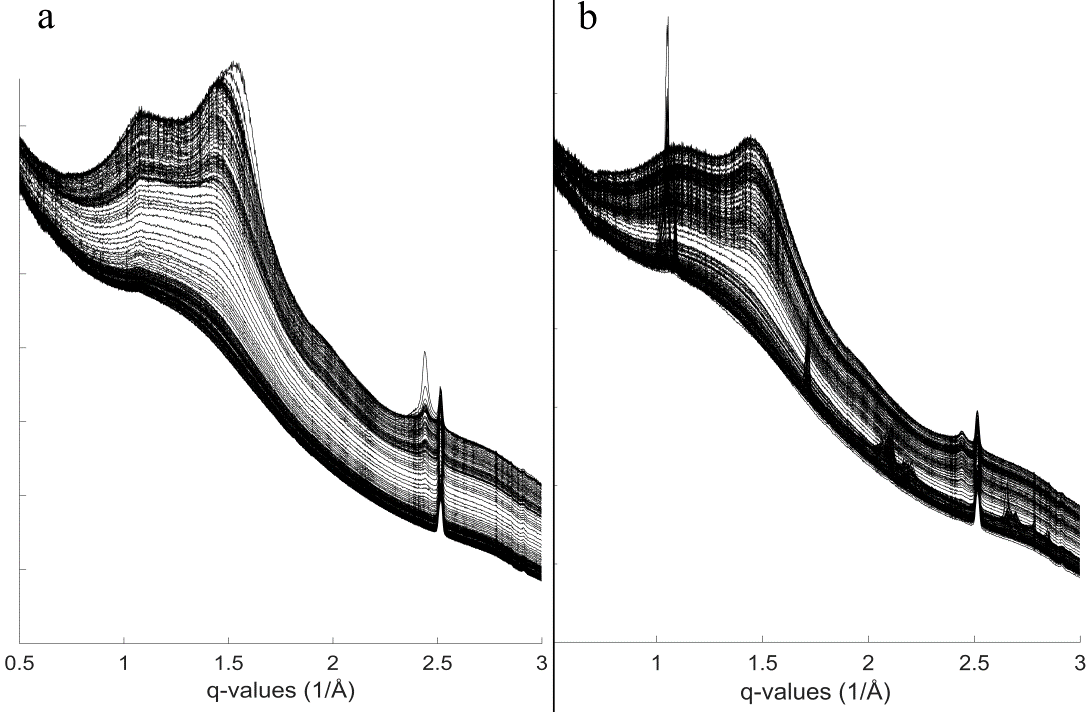


Figure S5. Examples of the radial profiles (the full 2D pattern integrated without any corrections in the data and printed every 1000^th^ of the data points) in TW vs OW. In (a), the radial profiles of TW in sample 11 are presented. For comparison, in (b), the radial profiles of OW in sample 12 are presented. Spike around q= 2.5 Å^-1^ arises from the used equipment and does not represent sample data.
